# Editorial: Biotechnology of phosphate solubilizing microorganisms for metabolites regulation: present and future

**DOI:** 10.3389/fbioe.2023.1258741

**Published:** 2023-08-02

**Authors:** Da Tian, Haoming Chen, Gilberto de Oliveira Mendes, Yanfang Feng

**Affiliations:** ^1^ Anhui Province Key Laboratory of Farmland Conservation and Pollution Prevention, College of Resources and Environment, Anhui Agricultural University, Hefei, China; ^2^ School of Environmental and Biological Engineering, Nanjing University of Science and Technology, Nanjing, China; ^3^ Laboratório de Microbiologia e Fitopatologia, Instituto de Ciências Agrárias, Universidade Federal de Uberlândia, Uberlândia, Brazil; ^4^ Key Laboratory of Agro-Environment in Downstream of Yangze Plain, Jiangsu Academy of Agricultural Sciences, Ministry of Agriculture and Rural Affairs of China, Nanjing, China

**Keywords:** biotechnology, heavy metal remediation, phosphate solubilizing microorganisms, phosphate dissolution, metabolism

Phosphate solubilizing microorganisms (PSM) can transform insoluble phosphate into plant-absorbable forms in soil by producing metabolites, including phosphate solubilizing fungi (PSF), phosphate solubilizing bacteria (PSB), and phosphate solubilizing actinomycetes. However, promoting the capacity of metabolism *via* biotechnology approaches is a current challenge for PSM. This paper overview the primary functional metabolites of PSM and the related biotechnology approaches to improve their application in agriculture and environmental remediation.

Sun et al. found manure is a potential substitute for mineral phosphorus (P) fertilizers, which can enhance the bacterial organic P mineralization and increase soil available P content. Jiang et al.overviewed the electrochemical technology for enhanced P solubilization by PSM. The application of this technology not only provides a suitable environment for the metabolism of PSM but also promotes the growth of PSM. Tian et al. overviewed lead (Pb) remediation by phosphate solubilizing fungi (PSF) and apatite. The primary pathway for the stable Pb minerals formation needs to enhance the production of oxalic acid by PSF. Pan et al. studied the physiological response of PSF under Pb and cadmium (Cd) stress *via* the technology of scanning electron microscopy and transmission electron microscopy. Compared with Cd, PSF has a higher Pb accumulation in both the extracellular and intracellular. Zhang et al. isolated a phosphate solubilizing bacteria (PSB) strain from saline-alkali soil. This PSB has high phosphate solubilizing capacity and can remove 90.8% Pb cations under a 500 mg/L Pb level. Chen et al. summarized that biochar can enhance PSM survival in a high heavy metal concentration. The biotechnology of biochar and PSM combination is a promising approach in heavy metal remediation. Wang et al. overviewed the technology of biofertilizers, soil conditioners, remediation agents, etc., which could be potential pathways to solve the PSM commercialization and application. Wu and Zhao summarized a new technology of machine learning in assessing the efficiency of PSM in phosphate dissolution and heavy metal remediation. In addition, Chen et al.found that the PSM can also apply to mine phytoremediation as a driving force. PSM can function in the establishment and development of plants and ecosystems, which has great potential to reduce heavy metal toxicity and promote the growth of plants.

This Research Topic generally reviews the metabolic characteristics of PSM and the biotechnology approaches to enhance metabolic production. These biotechnologies have promoted the application of PSM in agriculture and heavy metal remediation. Significantly, these potential biotechnologies could also extend the application of PSM in food fermentation and biocontrol of plant pathogens.

